# Experts prioritize osteoarthritis non-surgical interventions from Cochrane systematic reviews for translation into “Evidence4Equity” summaries

**DOI:** 10.1186/s12939-021-01477-4

**Published:** 2021-06-10

**Authors:** Elizabeth Houlding-Braunberger, Jennifer Petkovic, Nicholas Lebel, Peter Tugwell

**Affiliations:** 1 Clinical Epidemiology Program, Ottawa Hospital Research Institute, Ottawa, Canada; 2 Faculty of Science, University of Ottawa, Ottawa, Canada; 3 Department of Physical Therapy, Faculty of Medicine, University of Toronto, Toronto, Canada; 4 Bruyère Research Institute, University of Ottawa, Ottawa, Canada; 5 WHO Collaborating Centre for knowledge Translation and Health Technology Assessment in Health Equity, Bruyère Research Institute, Ottawa, Canada; 6 Department of Medicine, Faculty of Medicine, University of Toronto, Toronto, Canada; 7 Department of Medicine, Faculty of Medicine, University of Ottawa, Ottawa, Canada

**Keywords:** Osteoarthritis, Knowledge translation, Equity, Priority setting, Systematic reviews

## Abstract

**Objective:**

Osteoarthritis generates substantial health and socioeconomic burden, which is particularly marked in marginalized groups. It is imperative that practitioners have ready access to summaries of evidence-based interventions for osteoarthritis that incorporate equity considerations. Summaries of systematic reviews can provide this. The present study surveyed experts to prioritize a selection ofinterventions, from which equity focused summaries will be generated. Specifically, the prioritized interventions will be developed into Cochrane Evidence4Equity (E4E) summaries.

**Methods:**

Twenty-seven systematic reviews of OA interventions were found. From these, twenty-nine non-surgical treatments for osteoarthritis were identified, based on statistically significant findings for desired outcome variables or adverse events. Key findings from these studies were summarised and provided to 9 experts in the field of osteoarthritis.. Expert participants were asked to rate interventions based on feasibility, health system effects, universality, impact on inequities, and priority for translation into equity based E4E summaries. Expert participants were also encouraged to make comments to provide context for each rating. Free text responses were coded inductively and grouped into subthemes and themes.

**Results:**

Expert participants rated the intervention home land-based exercise for knee OA highest for priority for translation into an E4E summaries, followed by the interventions individual land-based exercise for knee OA, class land-based exercise for knee OA, exercise for hand OA and land-based exercise for hip OA. Upon qualitative analysis of the expert participants’ comments, fifteen subthemes were identified and grouped into three overall themes: (1) this intervention or an aspect of this intervention is unnecessary or unsafe**;** (2) this intervention or an aspect of this intervention may increase health inequities; and (3) experts noted difficulties completing rating exercise.

**Conclusion:**

The list of priority interventions and corresponding expert commentary generated information that will be used to direct and support knowledge translation efforts.

**Supplementary Information:**

The online version contains supplementary material available at 10.1186/s12939-021-01477-4.

## Introduction

Osteoarthritis (OA) is the most common form of arthritis and is associated with considerable physical, psychological, and socioeconomic burden [1, 2]. This financial burden disproportionately impacts individuals from lower socioeconomic demographic groups. Further, the rate of OA is significantly higher among disadvantaged populations. One scoping review, investigating rates of OA in a selection of high-income countries, found that individuals from a lower socioeconomic demographic had both higher rates of OA, and worse outcomes [3]. Living in a rural setting, being a member of a racialized community, being unemployed or being from a non-professional occupation have all been associated with increased risk for both OA rates and severity of symptoms [3–5]. A study investigating economic inequality among individuals reporting OA calculated a significantly higher burden of disease (OR = 15.1, 95% CI:11.4–20.0) in the poorest compared to the wealthiest cohorts [6]. Indigenous peoples in Australia or Canada also experience higher rates of OA [4, 7, 8]. Thus, it is particularly important to specifically investigate the implementation of OA interventions among disadvantaged populations in order to ensure maximum pain reduction and functional improvement.

Disparities also exist in access to non-surgical treatment for individuals with osteoarthritis. In addition to increased prevalence of OA among individuals in marginalized situations, access to non-surgical care is often reduced in these groups. A US study demonstrated that Hispanic minorities were less likely to have private medical insurance and were less likely to report using exercise to manage their OA in the last 6 months compared to non-Hispanic populations [9]. Another study reported that while there is little difference in self-care strategies between racial groups, older African American persons were significantly less likely to hold prescriptions for non-steroidal anti-inflammatory agents and instead more likely to use over-the-counter analgesics [10].. Further, African Americans were more likely to only have Veterans Affairs insurance only, although they were also less likely to report difficulty getting medical care when needed [11]. Lastly, while Indigenous populations in Canada, Australia, New Zealand and USA were 37–300% more likely to be hospitalized for osteoarthritis complications, they were 36–51% less likely to access specialized OA care, thus contributing to health outcome inequities [12]. These differences are concerning, considering failure to access treatment is associated with considerable costs and leads to the least amount of quality-adjusted life years (QALYs) gained [13]. Equitable implementation of osteoarthritis interventions could help prevent intervention-generated inequities and importantly increase the quality of life of disadvantaged individuals living with osteoarthritis [14].

The above noted socioeconomic variables and impacts on OA rates and interventions underline the importance of evaluating the value of current and new OA services from an equity-based perspective. However, single clinical studies, even high quality randomised controlled trials are insufficient to address health inequities and drive change in clinical practice [15–17]. Conducting comprehensive systematic reviews and then translating these reviews into accessible plain language summaries and guidelines is critical for successfully translating clinical science into improved health [18, 19]. Grimshaw 2012 states that addressing barriers to knowledge translation and health implementation is an essential step in this process [20]. One such barrier is the overwhelming volume of clinical evidence currently produced [21]. There are over 7900 systematic reviews within the Cochrane library alone. This large volume of reviews poses a barrier to health professionals who access the Cochrane library, especially considering reviews do not necessarily consider health inequities. A scoping review of interventions that improve health care quality for disadvantaged populations with OA reported a paucity of studies specifically commenting on reporting the applicability of interventions to vulnerable populations [3, 22]. O’Neill argues that increasing availability of an intervention within a country or region is not necessarily sufficient to reduce health inequities. The intervention must also be accessible, acceptable and effective in the most disadvantaged populations. Intervention-generated inequities can result in improvement of few, relatively advantaged individuals, but a lack of results in those who are more disadvantaged [14, 19].. Thus it is important to make information regarding the impact of intervention on inequity readily available to policy analysts and clinicians as to which interventions decrease or increase inequities.

The Campbell and Cochrane Equity Methods Group is dedicated to the translation and dissemination of evidence to decrease known and emerging health inequities. To this end, the E4E initiative was developed to prioritize and translate this enormous volume of systematic reviews into user-friendly summaries to improve access and applicability of interventions for disadvantaged populations [23]. E4E priority-setting exercises have already been applied to Cochrane reviews for treatment of depression, diabetes and obesity, HIV/AIDS, malaria, and nutrition [24]. Thus far, there has been no E4E summary of Cochrane reviews on osteoarthritis to direct equitable decision making among policy makers and health practitioners. There is a need to effectively identify which, of all the osteoarthritis interventions identified in the Cochrane library systematic reviews, should be translated for the E4E platform. In this study, 9 international experts in the field of osteoarthritis completed a priority setting exercise to determine which osteoarthritis interventions should be prioritized for subsequent development into equity-focused E4E plain language summaries.

## Methods

### Selection of interventions for inclusion in the priority-setting exercise

This priority-setting exercise was modeled from those previously used to complete E4E summaries on depression, diabetes and obesity, HIV/AIDS, malaria, and nutrition [24]. In order to identify relevant interventions for the survey, we conducted a search for systematic reviews of osteoarthritis treatments published in Cochrane Library from 2011 to 2018 using the search terms ‘osteoarthritis’ or ‘musculoskeletal’. This search generated a total list of 96 systematic reviews once duplicated were removed. These reviews were screened for pertinence to treatment of osteoarthritis and 69 records were excluded leaving 27 references whose full text was assessed for eligibility. Systematic reviews that did not report interventions were excluded. There were over 1211 outcomes for 149 unique interventions within these 27 reviews. We selected the interventions that had statistically significant findings for desired outcome variables or adverse events (using any measure). “Initially, the specific outcome variables were those prioritized by the Outcomes Measure in Rheumatology Initiative (OMERACT) [25], specifically any measure of pain, function, patient global assessment or quality of life. However, we decided to exclude function and patient global assessment from our list of outcomes: (1) in order to maximize participant response by providing a manageable number of interventions to rate (2) because there was substantial overlap in interventions with significant findings for pain and function. Where intervention studies reported multiple statistically significant findings in a time series (e.g. immediately post intervention; 3 months post intervention), we used the results from the longest time interval. Further, subgroup analyses were excluded as they were not consistently reported and thus not comparable across interventions. Where an intervention reported multiple outcomes that met inclusion criteria, these outcomes were all included. Using these criteria, a total of 34 outcomes for 29 unique interventions (extracted from 14 systematic reviews) were identified for inclusion in the priority-setting exercise provided to experts.

### Pre-data analysis preparation

In order to place findings from all interventions on a consistent metric, we converted all statistically significant effect sizes from the variables of interest into odds ratios using the formulae provided in the Cochrane Handbook [26]. Consistent with methodology in previous E4E projects [23], we converted negative effect sizes that demonstrated benefit to a positive value by reversal of the scale or taking the inverse of the OR. Interventions were ordered in a Word document according to converted effect size (OR), with most effective interventions placed first. As an exception, negative effects were listed alongside pain outcomes to provide experts with a holistic perspective of the efficacy and harms of an intervention. Quality of evidence was summarised as well. However, this was not provided to experts to remain consistent with previous rating exercises. This evidence was available on request and was provided to one expert who also requested that all interventions reporting the standardized mean difference be converted to mean differences. A sample of the final rating exercise can be found in Appendix A.

### Ratings

Respondents were asked to rate each intervention on five criteria, using a Likert scale (0–4). Criteria where chosen based on methodology from previous E4E projects [24]. Criteria were as follows;

#### Ease of implementation

This criterion referred to the ease with which the intervention can be implemented. Respondents were asked to consider if there was sufficient capacity to implement the intervention, and if the required staff training would be feasible. Ratings were from 0 = more difficult to 4 = optimal (easier to implement).

#### Health system requirements

This criterion referred to the impact the intervention would have on the health system. Respondents were asked to consider the level of difficulty with intervention delivery, the infrastructure required (human resources, facilities, etc.) and the affordability/ resources available within the broader health system. Ratings were from 0 = most/more difficult – 4 = optimal (easier/ fewer health system effects).

#### Universality/generalizability/share of burden

This criterion referred to the relevance of the intervention to a range of settings and people. Respondents were asked to consider whether the intervention was relevant to most countries, whether it posed any safety concerns, and whether these concerns may be different in different settings. Ratings were from 0 = less generalizable/ specific population - 4 = optimal (more generalizable/ population-based).

#### Impact on inequities

This criterion referred to the distribution of the disease burden in populations. Respondents were asked to consider whether the disadvantaged individuals were most likely to benefit from the intervention and whether the intervention could improve equity in long-term disease burden distribution. The characteristics of disadvantaged populations were defined according to the ‘PROGRESS +’ acronym (Place of residence, Race/ethnicity/culture/language, Occupation, Gender/sex, Religion, Education, Socioeconomic status, Social Capital, and Plus referring to personal characteristics associated with discrimination, features of relationships, and time-dependant relationships) [19]. Ratings were from 0 = increase inequities - 4 optimal (decrease inequities).

#### Overall rating

This criterion referred to the degree to which the intervention was important to prioritise in an E4E summary. Ratings were from 0 = least important and 4 = highest priority interventions.

#### Safety concerns

In addition, respondents were also able to indicate safety concerns for a given intervention (Y/N). Space was provided to comment on whether safety concerns would impact ratings.

### Participants

This priority-setting exercise was modeled from those previously used to complete E4E summaries on depression, diabetes and obesity, HIV/AIDS, malaria, and nutrition [23, 24]. Fourteen physicians, policy makers and research experts in the field of OA were identified and approached to participate in the study. As in previous exercises, experts were identified by members of the team. The majority (60%) of identified experts were involved in the OMERACT OA Working group [25], while others had equity- or musculoskeletal health- specific expertise. All had extensive experience with systematic reviews and were experts in the field of osteoarthritis. Ten of fifteen experts (67%), responded to an initial email invitation to participate in the study. Nine of the fifteen experts (60%) completed the priority-setting exercise, representing the final sample for the present study. Participants were mostly male (*n* = 6, 67%) and were employed as clinical researchers (*n =* 7, 78%) or researchers (*n* = 2, 22%). They were employed in Canada (*n* = 4, 44%), Australia (*n* = 3, 33%), the United Kingdom (*n* = 1, 11%) and France (*n* = 1, 11%).

Experts were invited to participate by email. Respondents were emailed a rating sheet with the compiled list of interventions, along with original effect sizes and their corresponding converted OR. They were asked to assign 5 ratings to each of 34 items (a given intervention and outcome measure) extracted from 14 Cochrane systematic reviews. Respondents also received a more detailed set of instructions with a brief comment on incidence and treatment of osteoarthritis in disadvantaged populations (Appendix A).

### Data analysis

#### Quantitative

Mean ratings on all five criteria for each of the interventions were generated (see Table 1). Consistent with previous E4E methodology [24], these ratings were converted to a score out of 100 for easier interpretations by reviewers (scores were divided by 4 and multiplied by 100). In order to further elucidate which criteria influenced the experts’ ‘overall’ score, Pearson correlation values were calculated for each of the four equity-based criteria (‘ease of implementation’, ‘health system requirements’, ‘universality’, and ‘impact on inequities’) using Excel. Linearity, homoscedasticity and absence of outliers was observed in scatterplots of the ratings for each criterion as a function of the overall ratings. Interventions that were rated according to different outcome measures were assigned one final score, calculated from an average of scores assigned to independent outcome measures.

**Table 1:** Ratings of bottom interventions

Intervention		Ease of implementation^a^	Health System Effects ^b^	Universality^c^	Impact on Inequities^d^	Overall Rating^e^
1	Home land-based exercise vs no exercise for knee OA	Range: 2–4 Total: 86.11 Rating: 1	Range: 2–4 Total: 75.00 Rating: 3	Range: 2–4 Total: 91.67 Rating: 1	Range: 1–4 Total: 72.22 Rating: 2	Range: 2–4 Total: 88.89 Rating: 1
2	Individual Land-based exercise vs no exercise for knee OA	Range: 2–4 Total: 86.11 Rating: 1	Range: 2–4 Total: 80.56 Rating: 2	Range: 2–4 Total: 91.67 Rating: 1	Range: 1–4 Total: 69.44 Rating: 3	Range: 2–4 Total: 86.11 Rating: 2
3	Class land-based exercise vs no exercise for knee OA	Range: 2–4 Total: 83.33 Rating: 3	Range: 2–4 Total: 72.22 Rating: 5	Range: 2–4 Total: 91.67 Rating: 1	Range: 1–4 Total: 66.67 Rating: 8	Range: 2–4 Total: 86.11 Rating: 2
4	Exercise vs no exercise for hand OA	Range: 2–4 Total: 83.33 Rating: 3	Range: 2–4 Total: 72.22 Rating: 5	Range: 2–4 Total: 91.67 Rating: 1	Range: 1–4 Total: 69.44 Rating: 3	Range: 2–4 Total: 86.11 Rating: 2
5	All land-based exercise vs no exercise for hip OA	Range: 2–4 Total: 81.00 Rating: 5	Range: 2–4 Total: 83.00 Rating: 1	Range: 1–4 Total: 81.00 Rating: 6	Range: 1–4 Total: 76.00 Rating: 1	Range: 2–4 Total: 86.00 Rating: 5
6	High vs low intensity exercise	Range: 2–4 Total: 75.00 Rating: 8	Range: 2–4 Total: 72.22 Rating: 5	Range: 2–4 Total: 86.11 Rating: 5	Range: 1–4 Total: 66.67 Rating: 8	Range: 2–4 Total: 80.56 Rating: 6
7	Self-Management Program vs usual care/no treatment/wait list	Range: 2–4 Total: 72.22 Rating: 9	Range: 2–4 Total: 72.22 Rating: 5	Range: 2–4 Total: 77.78 Rating: 7	Range: 1–4 Total: 69.44 Rating: 3	Range: 2–4 Total: 75.00 Rating: 7
8	Celecoxib vs placebo	Range: 2–4 Total: 81.00 Rating: 5	Range: 2–4 Total: 75.00 Rating: 3	Range: 2–4 Total: 72.00 Rating: 8	Range: 1–4 Total: 69.00 Rating: 6	Range: 2–4 Total: 69.00 Rating: 8
9	Ayurvedic RA-II vs placebo	Range: 0–4 Total: 61.00 Rating: 23	Range: 0–4 Total: 50.00 Rating: 25	Range: 0–4 Total: 58.00 Rating: 20	Range: 0–4 Total: 58.00 Rating: 15	Range: 0–4 Total: 61.00 Rating: 9
10	Chondroitin sulfate (+ glucosamine) vs placebo or control ≥800 mg/d	Range: 0–4 Total: 72.00 Rating: 12	Range: 0–4 Total: 64.00 Rating: 10	Range: 0–4 Total: 56.00 Rating: 25	Range: 0–4 Total: 56.00 Rating: 19	Range: 0–4 Total: 56.00 Rating: 10
11	Corticosteroid vs sham injection/no treatment	Range: 1–4 Total: 58.00 Rating: 27	Range: 1–4 Total: 61.00 Rating: 14	Range: 1–4 Total: 61.00 Rating: 12	Range: 1–4 Total: 67.00 Rating: 7	Range: 1–4 Total: 56.00 Rating: 10
12	Lateral wedge insole versus no insole	Range: 0–4 Total: 61.11 Rating: 12	Range: 0–4 Total: 58.33 Rating: 10	Range: 0–4 Total: 63.98 Rating: 7	Range: 0–4 Total: 52.78 Rating: 11	Range: 0–4 Total: 55.56 Rating: 11
13	Aquatic exercise vs control (usual care, education, social attention, telephone call, waiting list for surgery) immediately after treatment: knee and hip OA	Range: 1–4 Total: 56.00 Rating: 16	Range: 1–4 Total: 57.00 Rating: 11	Range: 2–4 Total: 64.00 Rating: 6	Range: 1–3 Total: 51.00 Rating: 12	Range: 2–3 Total: 54.00 Rating: 12
14	Chondroitin vs Placebo ≥800 mg/d	Range: 0–4 Total: 72.22 Rating: 6	Range: 0–4 Total: 69.44 Rating: 5	Range: 0–4 Total: 52.78 Rating: 14	Range: 0–4 Total: 52.78 Rating: 11	Range: 0–4 Total: 52.78 Rating: 13
15	Brace vs no treatment	Range: 1–4 Total: 58.33 Rating: 14	Range: 0–4 Total: 47.22 Rating:17	Range: 0–4 Total: 58.33 Rating: 10	Range: 0–4 Total: 52.78 Rating: 11	Range: 0–4 Total: 50.00 Rating: 14
16	*Harpagophytum procumbens* vs diacerhein	Range: 0–4 Total: 63.89 Rating: 11	Range: 0–4 Total: 58.33 Rating: 10	Range: 0–4 Total: 61.11 Rating: 8	Range: 0–4 Total: 58.33 Rating: 7	Range: 0–4 Total: 50.00 Rating: 14
17	*Boswellia serrata*, enriched (100 mg) + non-volatile oil vs placebo	Range: 0–4 Total: 66.67 Rating: 9	Range: 0–4 Total: 52.78 Rating: 15	Range: 0–4 Total: 58.33 Rating: 10	Range: 0–4 Total: 58.33 Rating: 7	Range: 0–4 Total: 47.22 Rating: 15
18	Acupuncture + routine vs routine alone	Range: 1–4 Total:58.33 Rating: 14	Range: 0–4 Total: 55.56 Rating: 13	Range: 0–4 Total: 55.56 Rating: 13	Range: 0–4 Total: 50.00 Rating: 13	Range: 0–4 Total: 47.22 Rating: 15
19	Reumalex vs placebo	Range: 0–4 Total: 66.67 Rating: 9	Range: 0–4 Total: 52.78 Rating: 15	Range: 0–4 Total: 58.33 Rating: 10	Range: 0–4 Total: 58.33 Rating: 7	Range: 0–4 Total: 47.22 Rating: 15
20	Persea gratissma + *Glycine max* (600 mg) vs placebo	Range: 2–4 Total: 72.22 Rating: 6	Range: 0–4 Total: 61.11 Rating: 8	Range: 0–4 Total: 58.33 Rating: 10	Range: 0–4 Total: 58.33 Rating: 7	Range: 0–4 Total: 47.22 Rating: 15
21	Acupuncture vs sham acupuncture	Range: 0–4 Total: 58.33 Rating: 14	Range: 0–4 Total: 47.22 Rating: 17	Range: 0–3 Total: 58.33 Rating: 10	Range: 0–4 Total: 50.00 Rating: 13	Range: 0–4 Total: 47.22 Rating: 15
22	*Pinus pinaster* (150 mL) vs placebo	Range: 0–4 Total: 67.00 Rating: 8	Range: 0–4 Total: 53.00 Rating: 14	Range: 0–4 Total: 58.00 Rating: 11	Range: 0–4 Total: 58.00 Rating: 8	Range: 0–4 Total: 47.00 Rating: 16
23	SKI306X (1800 mg) vs placebo	Range: 0–4 Total: 67.00 Rating: 8	Range: 0–4 Total: 56.00 Rating: 12	Range: 0–4 Total: 58.00 Rating: 11	Range: 0–4 Total: 58.00 Rating: 8	Range: 0–4 Total: 47.00 Rating: 16
24	Boswellia serrata, enriched (100 mg) vs placebo	Range: 2–4 Total: 72.00 Rating: 7	Range: 0–4 Total: 53.00 Rating: 14	Range: 0–4 Total: 58.00 Rating: 11	Range: 0–4 Total: 58.00 Rating: 8	Range: 0–4 Total: 47.00 Rating: 16
25	*Salix purpurea* x daphnoides vs diclofenac	Range: 1–4 Total: 76.39 Rating: 4	Range: 0–4 Total: 63.89 Rating: 7	Range: 0–4 Total: 58.33 Rating: 10	Range: 0–4 Total: 55.56 Rating: 10	Range: 0–4 Total: 44.44 Rating: 17
26	Hyaluronic acid vs placebo + progressive ankle exercise	Range: 0–3 Total: 47.22 Rating: 17	Range: 1–4 Total: 47.22 Rating: 17	Range: 0–3 Total: 50.00 Rating: 15	Range: 0–4 Total: 44.44 Rating: 15	Range: 0–4 Total: 44.44 Rating: 17
27	*Zingiber officinale* + *Alpinia galanga* (EV.EXT77) vs placebo	Range: 0–4 Total: 66.67 Rating: 9	Range: 0–4 Total: 61.11 Rating: 8	Range: 0–4 Total: 58.33 Rating: 10	Range: 0–4 Total: 58.33 Rating: 7	Range: 0–4 Total: 44.44 Rating: 17
28	Acupuncture vs NSAIDs	Range: 1–4 Total: 64.00 Rating: 10	Range: 0–4 Total: 47.00 Rating: 18	Range: 0–4 Total: 58.00 Rating: 11	Range: 0–4 Total:47.00 Rating: 14	Range: 0–3 Total: 42.00 Rating: 18
29	Opioids versus placebo	Range: 0–4 Total: 67.00 Rating: 8	Range: 0–4 Total: 56.00 Rating: 12	Range: 0–4 Total: 56.00 Rating: 12	Range: 0–4 Total: 43.00 Rating: 16	Range: 0–3 Total: 28.00 Rating: 19

^a^For Ease of Implementation: How easily can the intervention be added to usual care without requiring too much effort from health workers or disrupting practice. Consider how much effort is required, what training and resources are needed, what kind of scheduling and follow up, physical space, etc. Ratings are 0 to 4. 4 = optimal (easier to implement), 0 = more difficult. Total score has been converted to a score out of 100

^b^For Health System Effects: the impact the intervention will have on the health system. Certain interventions may be easy to implement but will still have large system effects – they may require incremental or major changes to the health system. 4 = optimal (easier/fewer health system effects), 0 = more difficult/greater health system effects. Total score has been converted to a score out of 100

^c^For universality: Applicable and beneficial to large numbers including interventions targeted at one segment of the population (e.g. men) but the effects are wider than those targeted for the intervention (e.g. circumcision of men helps prevent HIV infection in women). An intervention targeted to all the PLHIV should be considered over for example MSM’s living with HIV who have TB and Hep C co-infection - a very small specific group. Ratings are 0 to 4. 4 = Optimal (more generalizable/population-based, 0 = less generalizable/specific population. Total score has been converted into a score out of 100

^d^Does the distribution of the disease burden affect mainly the disadvantaged? Are the disadvantaged most likely to benefit from the intervention? Will the intervention improve equity in disease burden distribution long-term? Ratings are 0–4. 4 = optimal interventions that would decrease inequities. 0 = interventions that may increase inequities. Total score has been converted to a score out of 100

^e^Overall rating indicate prioritization for knowledge translation into an E4E summary. 4 = high priority for knowledge translation into an E4E summary. 0 = low priority for knowledge translation into an E4E summary. Total score has been converted to a score out of 100

#### Qualitative

Expert’s free text responses were coded using an inductive approach as described in previous studies [27–29] wherein the content of the responses was used to develop a coding framework. Two independent coders (EH, NL) developed separate coding schema, and then discussed and settled disagreements by consensus. These same two coders completed coding separately. Percent discordances were calculated, and final decisions were made by consensus. The interventions and corresponding comments were grouped together according to the Cochrane systematic review from which they were extracted to concisely summarise the large number of comments (Appendix B). To account for systematic reviews with many similar interventions with comments repeated multiple times, we calculated the proportion of comments made for each systematic review as a function of the number of interventions included in the exercise. Individual codes were grouped inductively into themes and subthemes.

## Results

**Fig. 1:**
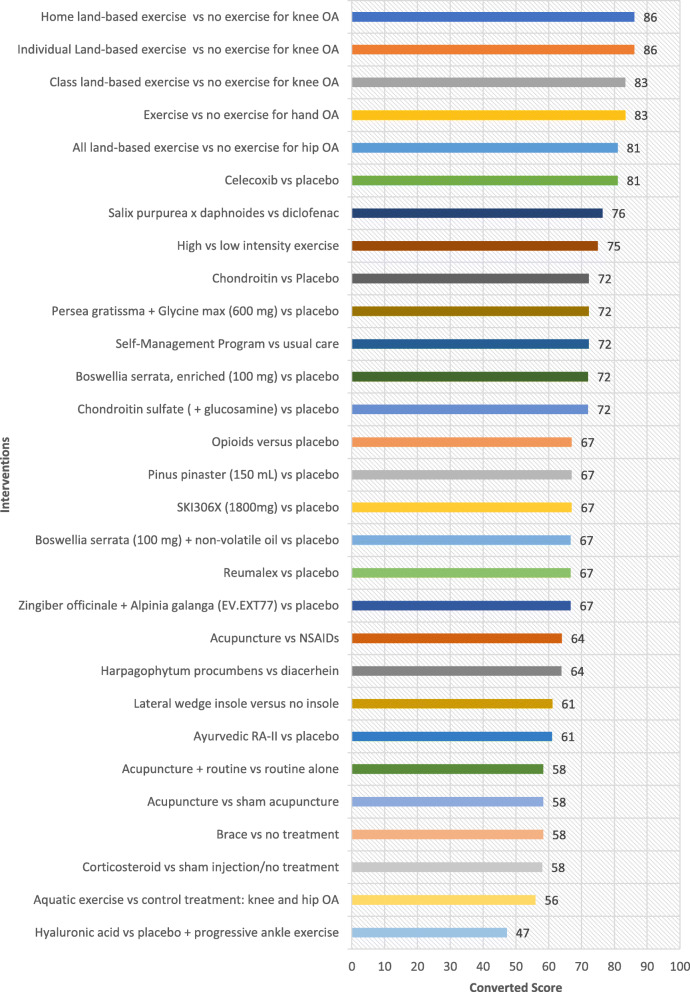
Ratings for Ease of Implementation

**Fig. 2:**
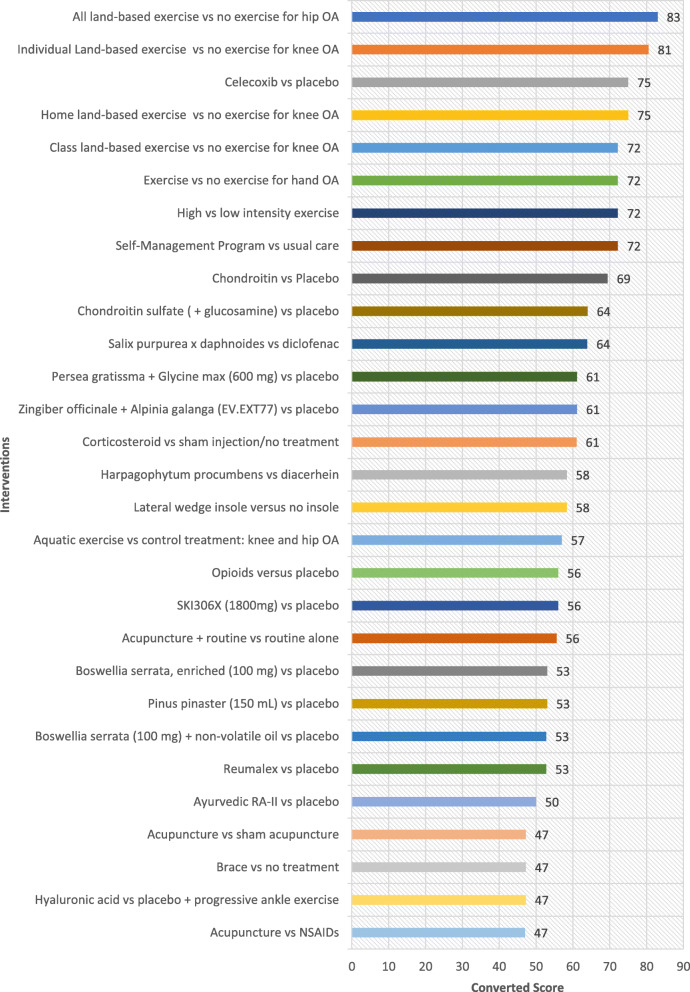
Ratings for Health System Effects

**Fig. 3:**
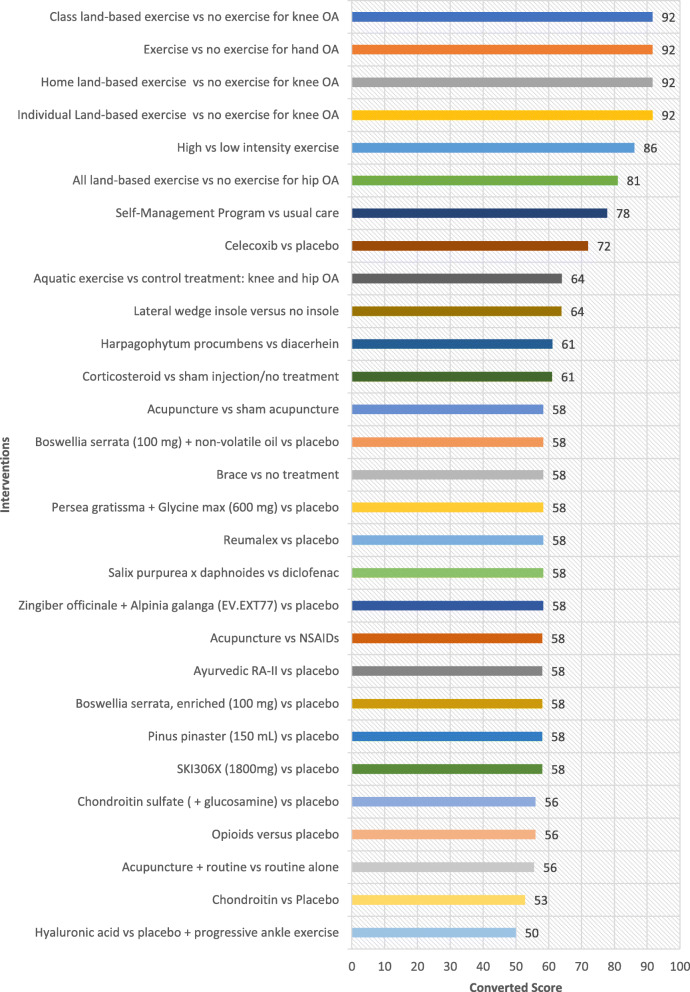
Ratings for Universality

**Fig. 4:**
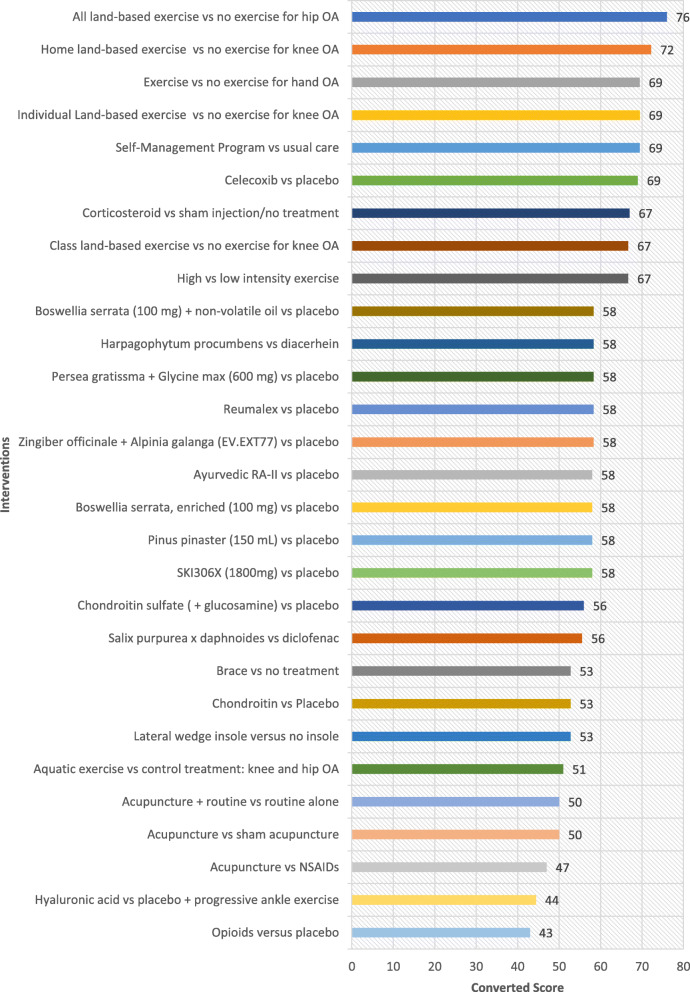
Ratings for Impact on Inequities

**Fig. 5:**
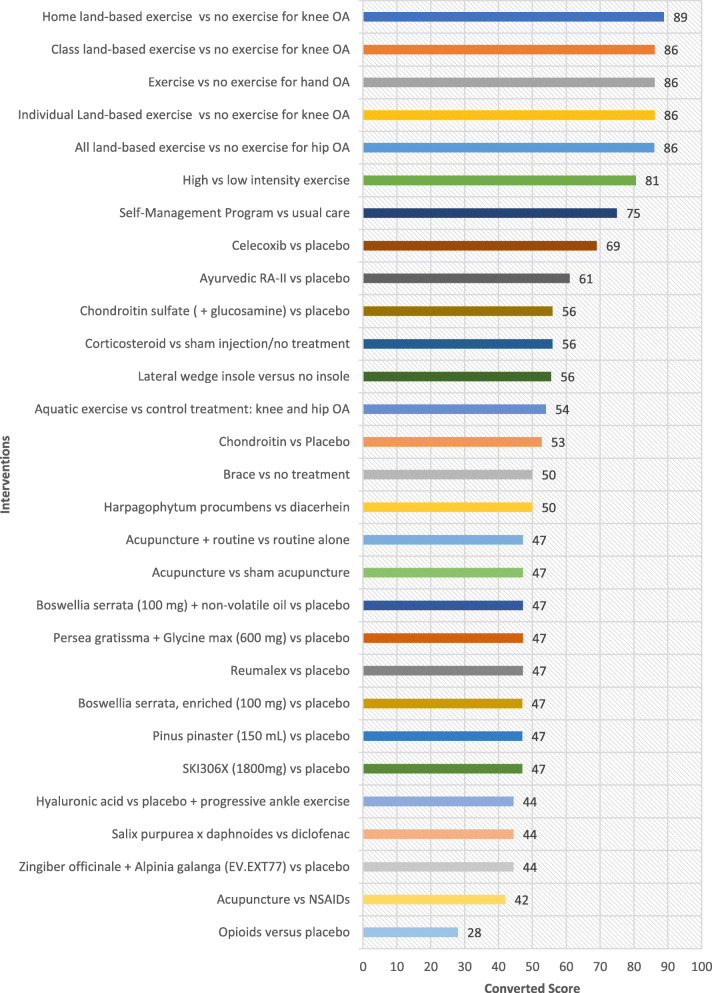
Ratings Overall for Translation into an E4E Summary

Table 1 shows all interventions ordered by the highest ratings in the ‘Overall Priority’ category. These ratings indicate which interventions experts perceive merit translation based on their potential to improve the health of individuals who are disadvantaged, when ease of implementation, health system requirement, universality and impact on inequities are considered. Ordered lists based on the highest rated interventions for each of the other five criteria are provided as well (Fig. 1, 2, 3, 4 and 5). The top 5 interventions were as follows: (1) “Home land-based exercise vs no exercise for knee OA”, (2) “Class land-based exercise vs no exercise for knee OA”, (3) “Exercise or no exercise for hand OA”, (4) “Individual land-based exercise vs no exercise for knee OA” and (5) “All land-based exercise vs no exercise for hip OA”. The top 11 interventions prioritized overall were extracted from the following systematic reviews: Exercise for osteoarthritis of the knee [30], Exercise for hand osteoarthritis [31], Exercise for osteoarthritis of the hip [32], High-intensity versus low-intensity physical activity or exercise in people with hip or knee osteoarthritis [33], Self-management education programmes for osteoarthritis [34], Celecoxib for osteoarthritis [35], Oral herbal therapies for treating osteoarthritis [36], Chondroitin for osteoarthritis [37], and Intra-articular corticosteroid for knee osteoarthritis [38]. The top six interventions involved treatment of OA with exercise (four specifying land-based exercise) and the 7th intervention involved self-management programs for treatment of OA (Table 1). On the whole, experts gave higher ratings to interventions treating OA with exercise and lower ratings to pharmacological interventions including opioids, oral herbal therapies, celecoxib, glucosamine and chondroitin sulfate. Notably, aquatic exercise was lower rated compared to land-based exercise. To further elucidate which criteria may have influenced the expert’s ‘Overall’ ratings, each of the four equity-focused criteria (‘Ease of Implementation’, ‘Health System Requirements’, ‘Universality’, and ‘Impact on Inequities) was plotted against the ‘Overall’ criterion measuring expert opinion of priorities for an E4E summary. ‘Universality’ was estimated to have the greatest impact on respondents ‘Overall’ rating with a Pearson correlation of 0.96. This was greater than for the criteria ‘Ease of implementation’, ‘Health system requirements’ and ‘Impact on inequities’ which had Pearson correlation values of 0.82, 0.85 and 0.84 respectively. These values were all statistically significant (alpha< 0.05).

### Safety concerns

Based on suggestions from previous priority-setting exercises used to develop E4E summaries [24], experts were invited to identify safety concerns associated with rated interventions. A total of 17 (58.6%) interventions were flagged for safety concerns by at least one reviewer. None of the top 7 prioritized interventions had any safety concerns. However, experts identified safety concerns for all pharmacological interventions as well as those included in the top 11 list of interventions rated highest for ‘Overall Priority’.

### Qualitative analysis

Following qualitative coding we identified three themes overall which were further divided into subthemes.

#### Theme 1 - this intervention or an aspect of this intervention is unnecessary or unsafe

The most frequent concerns brought forward by experts related to the overall safety of an intervention. The most common safety concerns were that an intervention was not effective and thus unnecessary, followed by comments that an intervention could increase a patient’s risk of adverse events, was not recommended by clinical practice guidelines, or required ongoing patient monitoring. Also noted were concerns regarding the risk of developing an addiction, infection risk from needle usage, the need to ‘pace-up’ the interventions, the need for greater quality control measures, and issues regarding patient compliance.

##### Subtheme 1.1: this intervention has questionable clinical efficacy

Fourteen comments noted that interventions had questionable clinical efficacy. Of these, five came from a review of oral herbal therapies, two from a review of braces and orthoses, two from a review of acupuncture, two from a review of chondroitin, and the remainder from reviews on hyaluronic acid, opioids, and intra-articular corticosteroids. One expert stated “[Mean difference] not clinically relevant” for three different oral herbal therapies, two different brace/orthotic interventions, and two different acupuncture interventions. This was important to capture as we excluded interventions with effect sizes that were not statistically significant but sought out expert opinion regarding the clinical relevance of effect sizes.

##### Subtheme 1.2: general safety concerns

There were 18 comments made regarding general safety concerns, of which 14 were from a review of oral herbal therapies (comprising 10 different interventions), two from a review of intra-articular corticosteroid for knee osteoarthritis, one from a review of celecoxib, and one from a review of high-intensity vs low-intensity physical activity. One expert expressed concerns regarding the “safety of [the] agent” for each of the 10 interventions in the oral herbal therapy review. For the review of intra-articular corticosteroids, one expert mentioned that the intervention could “damage [knee] cartilage”. When assessing the safety of high intensity exercise one expert noted there is a risk of “pain flares” and a need to administer exercise with “comorbidities” in mind. Lastly, regarding a review of celecoxib for osteoarthritis, an expert stated the risk of “adverse events” as a concern.

##### Subtheme 1.3: clinical practice guidelines conditionally, are neutral or do not recommend this intervention

One expert made 13 comments related to clinical practice guidelines and whether interventions were neutral, conditionally, or not recommended. Of these comments, four were from a review of oral herbal therapies, three from a review of acupuncture for hip osteoarthritis, two from a review of braces and orthoses, two from a review of chondroitin, one from a review of intra-articular corticosteroids, and one from a review of self-management programs. Of the comments made of oral herbal therapies, *Salix purpurea* was listed as ‘do not recommend’ whereas *Persea gratissima* and *Boswellia serrata* were cited as neutral. Regarding acupuncture, the expert suggested “not offering acupuncture for people with knee and/or hip OA” according to the current clinical practice guidelines. Concerning braces and orthoses for osteoarthritis, the same expert stated, “Clinical practice guidelines [are] conditional [ly] against this recommendation for lateral wedge medial tibial OA [and] neutral for lateral.” indicating that certain conditions need to be met before this intervention could be recommended. For intra-articular corticosteroids, the expert also mentioned that the clinical practice guidelines recommend that this intervention be “only [administered] as indicated when no progress [occurs] with other first line interventions.” Further, they stated, “CPGs conditional for recommendation” which again captures the idea that certain conditions should be met before administering this intervention. Further, they note that this intervention could increase inequity and requires “Simple messaging to mitigate health literacy issues (hence rating on inequity lower).”

##### Subtheme 1.4: this intervention requires ongoing monitoring (bp, side effects)

There were 5 comments made regarding the need for ongoing patient monitoring or broader health implementation requirements to support OA interventions: two from a review of opioids, two from a review of celecoxib, and one from a review of oral herbal therapies. One expert observed that there is “need [for] systems in place for safe use of opioids”. Regarding the use of celecoxib, experts commented that there is a “need to manage comorbidities risk” as well as a “need for monitoring cr [creatine rise] BP”. For oral herbal therapies, one expert mentioned that “[for] diclofenac - assess for GI bleeding risk, CVD risk and renal risks.”

##### Subtheme 1.5: there is a risk of addiction associated with this intervention and/or drug-trafficking

All 4 comments concerning the risk of addiction were made regarding a review on the use of opioids for osteoarthritis. Experts commented, “Risk of opioid addiction & traffic”, “Addiction [and] adverse events”, “Adverse outcomes with prolonged use; tolerance and addiction risks”, and simply, “Addiction.”

##### Subtheme 1.6: interventions that require use of needles run a risk of infection

Of the five comments made concerning the risk of infection from needles, two were from a review of acupuncture for hip osteoarthritis, two from a review of intra-articular corticosteroid, and one from a review on hyaluronic acid and other conservative treatment options for osteoarthritis of the ankle.

##### Subtheme 1.7: this intervention may need to be ‘paced up’ or implemented gradually

All five comments regarding the need to ‘pace-up’ interventions were made by one expert concerning three different reviews on the use of exercise to treat osteoarthritis: Exercise for osteoarthritis of the knee, Exercise for osteoarthritis of the hip, and High-intensity versus low-intensity physical activity or exercise in people with hip or knee osteoarthritis.

##### Subtheme 1.8: certain interventions require quality control measures to ensure integrity and safety of components

There were 20 comments made regarding the need for better quality control measures, of which 18 were from a review on oral herbal therapies and two from a review on chondroitin. Experts frequently mentioned either “quality control” or “consistency of components” for herbal therapies, referring to the need to ensure rigorous quality control, which is particularly important for herbal therapies regulated as natural health products.

##### Subtheme 1.9: when implementing this intervention, there may be difficulties associated with compliance

Finally, there was one comment regarding issues with patient compliance from a review on exercise for osteoarthritis of the knee.

#### Theme 2 - this intervention or an aspect of this intervention may increase health inequities

Following safety concerns, experts also frequently commented that certain interventions may potentially increase health inequities. The high cost of certain interventions in addition to a lack of access to the materials and/or expertise required to utilize such interventions were cited as the main sources driving inequities. Some experts also mentioned that certain interventions required adaptations to the cultural and social contexts in which they are found.

##### Subtheme 2.1: this intervention requires specific expertise which may not be accessible and may only be available in certain regions

Experts made 13 comments regarding the need for specific expertise in order to utilize interventions, of which seven came from five different reviews on exercise for osteoarthritis, two from a review on braces and orthoses for treating osteoarthritis of the knee, and one comment from reviews on oral herbal therapies, acupuncture, hyaluronic acid, and self-management education programs respectively. Regarding exercise for osteoarthritis, one expert stated that “[the patient] could attend community-based centers or gyms” which may require the use of personal trainers and other expertise that may not always be available in certain regions, such as rural and remote communities. Concerning the use of braces and orthoses for osteoarthritis, one expert mentioned that there is a need for the “Skill of [a] practitioner and availability of equipment.”

##### Subtheme 2.2: the material resources for this intervention may be difficult to access and/or is only available in certain regions

Experts made 12 comments regarding the requirement for materials only available in certain regions and/or difficult to source. Half of these comments were from three different reviews on exercise for osteoarthritis, two from a review on acupuncture, and the remainder from reviews on oral herbal therapies, braces and orthoses, and opioids.

##### Subtheme 2.3: this intervention is costly, may not be feasible in LMICs and may not be covered by insurance

Experts made 19 comments regarding the high cost of certain interventions. 10 of these comments were associated with a review on oral herbal therapies, two from a review on celecoxib, two from a review on acupuncture for hip osteoarthritis, two from a review on braces and orthoses, two from a review on chondroitin, and one from a review on hyaluronic acid. For 9 of the 10 interventions assessed in the review on oral herbal therapies, one expert commented that “Herbal therapies conditioned are costly in most countries”, highlighting the cost-inequities that many LMICs experience.

##### Subtheme 2.4: this intervention requires adaptations to cultural and social context

Experts made three comments regarding the need to adapt certain interventions to the social and cultural context in which they are used. One of these comments came from a review of self-management educational programs for osteoarthritis while the other came from a review of braces and orthoses for treating osteoarthritis of the knee. Regarding self-management educational programs, an expert stated “Need SMP adapted to cultural and social context”, while another commented “Simple messaging to mitigate health literacy issues (hence rating on inequity lower); digital or community-based dissemination.” One expert mentioned that “insoles [are] not practical or effective in countries where people go barefoot or in sandals.”

#### Theme 3 - experts noted difficulties completing rating exercise

Experts also brought up that it was difficult to complete the rating exercise for certain interventions due to concerns regarding their clinical efficacy as well as potential adverse outcomes. Regarding opioids, one expert commented that it was “Difficult to rate when outcome worse.” as this systematic review reported a greater number of adverse events among patients administered opioids compared with those administered a placebo.

## Discussion

Expert opinion suggests that translation of interventions involving exercise to treat osteoarthritis is more likely to reduce health inequities among disadvantaged populations with osteoarthritis than other non-surgical interventions. The six interventions rated highest in the ‘overall’ category all pertained to treatment of osteoarthritis with exercise programs. The seventh highest rated intervention was self-management programs for treatment of osteoarthritis. Experts gave most favourable ratings to interventions that promoted patient responsibility and self-management as opposed to pharmacological interventions that require prescription and monitoring for potentially harmful side effects. Land-based exercise completed at home was rates higher than class-based exercise completed in a gym or community centre. This again illustrates expert preference for interventions that are most convenient for patients to complete on their own. A notable exception to the prioritization of exercise-based interventions was aquatic exercise for osteoarthritis, which was rated in the middle at around the 13th position of the 29 interventions ‘overall’. However, this further reinforces that experts acknowledged that that regional or financial barriers could limit pool access (Table 2). Experts also commented that acupuncture for osteoarthritis may be limited and costly in non-Asian countries. Hesitation of experts to prioritize opioids may reflect the ongoing opioid epidemic [39–41], with some experts listing ‘addiction’ as a safety concern. ‘Universality’ may have had a greater influence on respondents’ likelihood to prioritize an intervention for knowledge translation than ‘Impact on equity’ which could indicate a preference among experts to guve higher ratings to interventions that work for many populations rather thanthose that are tailored for minority groups. This is consistent with trends observed in ‘overall’ ratings. If responses from this priority-setting exercise are used to direct knowledge translation efforts, there is still a need to monitor use of these interventions in order to determine if they are effective in reducing health inequities. Lastly, some respondents flagged interventions with effect sizes that were not clinically relevant or not supported by high quality evidence despite meeting our inclusion criteria of being statistically significant (Table 2). These comments were generally reflected in lower ratings (almost no oral herbal therapies and acupuncture made the top 11 list of interventions). There was one exception: 1. Ayurvedic RA-II vs placebo was rated 9 ‘overall’. This may have been due to the very large converted OR value for this intervention. While some experts commented that granular interventions such as oral herbal therapies should have been grouped together for easier interpretation, it is possible that no oral herbal therapies would have been prioritized in the top interventions if they were rated together, as most oral herbal therapies were rated quite low (Fig. 5).

**Table 2:** Themes developed in content analysis of expert comments

Theme	Subtheme
Theme 1 - This intervention or an aspect of this intervention is unnecessary or unsafe	1.1: This intervention has questionable clinical efficacy
	1.2: General safety concerns
	1.3: Clinical practice guidelines conditionally, are neutral or do not recommend this intervention
	1.4: This intervention requires ongoing monitoring (bp, side effects)
	1.5: There is a risk of addiction associated with this intervention and/or drug-trafficking
	1.6: Interventions that require use of needles run a risk of infection
	1.7: This intervention may need to be ‘paced up’ or implemented gradually
	1.8: Certain interventions require quality control measures to ensure integrity and safety of components
	1.9: When implementing this intervention, there may be difficulties associated with compliance
Theme 2 - This intervention or an aspect of this intervention may increase health inequities	2.1: This intervention requires specific expertise which may not be accessible and may only be available in certain regions
	2.2: The material resources for this intervention may be difficult to access and/or is only available in certain regions
	2.3: This intervention is costly, may not be feasible in LMICs and may not be covered by insurance
	2.4: This intervention requires adaptations to cultural and social context
Theme 3 - Experts noted difficulties completing rating exercise	–

### Limitations

The selection of experts could limit the generalizability of these results. Five of the experts were physicians and two of the nine experts were physiotherapists which could cause them to prioritize pharmacological and physical therapy interventions respectively for development into an E4E summary. However it is also possible that the professional backgrounds of experts might better inform their ratings. For instance, those who have worked as clinicians might have more accurate perceptions of issues related to accessibility/ inequity. A different selection of experts could have produced a different order of prioritized systematic review interventions. However, as the group of interventions prioritized overall is more relevant than the specific order, it is likely that a range of pertinent interventions outside of expert specialities was captured. While experts made some minor critiques regarding the methodology of the priority setting exercise, this was expected and solicited throughout communications with respondents. Overall, experts expressed confidence in their ability to rate interventions and effectively communicate which should be prioritized for an E4E summary.

## Conclusion

Cochrane publishes an increasing number of systematic reviews each year and aims to disseminate the latest in best practice care to health practitioners and combat a lack of replicability that occurs with even very large single studies [16, 17]. This is part of a trend of increased production of systematic reviews, from about 80 a year in the late 1980’s to almost 8000 a year globally in 2015 [18]. As systematic reviews have a significant impact on both development of health policy and practice [42] it is increasingly important to increase accessibility of current evidence through translation with an equity focus [43]. E4E summaries help to inform physicians and policy experts on how interventions increase or decrease the burden of disease specific to disadvantaged individuals. Through this priority setting exercise, experts selected systematic review interventions that should be summarised into E4E summaries as well as rating them for other criteria including universality and equity. Decisions regarding what interventions to prioritise for E4E summaries should take into consideration the concerns raised regarding interventions. For instance, only including interventions whose outcomes have clinically significant mean differences and that are relatively inexpensive in terms of financial cost, expertise required and ongoing monitoring. Based on the result of the present study we recommend that the interventions (1) “Home land-based exercise vs no exercise for knee OA”, (2) “Class land-based exercise vs no exercise for knee OA”, (3) “Exercise or no exercise for hand OA”, (4) “Individual land-based exercise vs no exercise for knee OA” and (5) “All land-based exercise vs no exercise for hip OA”.be prioritized overall for translation into an E4E summary. When developing these equity-focused summaries, we recommend the integration of experts’ comments noting that these exercise-based interventions need to be paced up, require additional education, require management of comorbidities, and require simple messaging to mitigate health literacy issues.

## Supplementary Information

**Additional file 1.**

**Additional file 2.**

## Data Availability

Please contact the author for data requests.
