# Supplementary material for: Experts prioritize osteoarthritis non-surgical interventions from Cochrane systematic reviews for translation into “Evidence4Equity” summaries
Source: Int J Equity Health. 2021 Jun 10;20:136. doi: 10.1186/s12939-021-01477-4 (PMC8193871; doi:10.1186/s12939-021-01477-4)
Supplement: Supplementary file 1 — Additional file 1. [file 12939_2021_1477_MOESM1_ESM.docx]

| ID | Review Title | Intervention | Outcome | Original effect size | Effect Size | Favours? | Ease of implementation^[[1]](#footnote-1)^  (0-4) | Health System Effects ^[[2]](#footnote-2)^  (0-4) | Universality ^[[3]](#footnote-3)^  (0-4) | Impact on Inequities^4^ (0-4) | Overall rating  (0-4) | Safety Concerns^5^ (Y/N) | Comments |
| --- | --- | --- | --- | --- | --- | --- | --- | --- | --- | --- | --- | --- | --- |
| 1 | Oral herbal therapies for treating osteoarthritis | Ayurvedic RA-II vs placebo | Pain (VAS) 0-100 | MD  -1.03 [-1.18, -0.88] | 164.50 | ***favours RA-II*** |  |  |  |  |  |  |  |
| 2 | Braces and orthoses for treating osteoarthritis of the knee | Brace vs no treatment | Pain (VAS)  9 months | MD  -2.80 [-3.58, -2.02] | 52.24 | ***favours brace*** |  |  |  |  |  |  |  |
| 4 | Oral herbal therapies for treating osteoarthritis | Boswellia serrata (enriched) 100 mg plus non-volatile oil vs placebo | Pain (VAS)  90 days | MD  -18.10 [-24.95, -11.25] | 19.77 | ***favours Boswellia serrata*** |  |  |  |  |  |  |  |
| 5 | Oral herbal therapies for treating osteoarthritis | Pinus pinaster (Pycnogenol® 150 mg) vs placebo | Pain (WOMAC-VAS) | MD  -142.0 [-199.55, -84.45] | 16.85 | ***favours Pycnogenol*** |  |  |  |  |  |  |  |
| 6 | Oral herbal therapies for treating osteoarthritis | Harpagophytum procumbens vs diacerhein | Pain (VAS) 0-100 change from baseline  120 days | MD  -5.10 [-6.52, -3.68] | 14.16 | ***favours Harpagophytum*** |  |  |  |  |  |  |  |
| 7 | Braces and orthoses for treating osteoarthritis of the knee | Lateral wedge insole versus no insole | Pain (VAS) | MD  -1.60 [-2.31, -0.89] | 11.98 | ***favours Lateral-wedge insole*** |  |  |  |  |  |  |  |
| 8 | Oral herbal therapies for treating osteoarthritis | SKI306X (1800mg) vs placebo | Pain (VAS) 0-100 change from baseline | MD  -22.3 [-31.82, -12.78] | 11.18 | ***favours SKI306X*** |  |  |  |  |  |  |  |
| 9 | Acupuncture for hip osteoarthritis | Acupuncture as addition to routine primary physician care vs routine primary physician care alone | Pain | MD  -22.90 [ -29.22, -16.58] | 8.97 | ***favours acupuncture*** |  |  |  |  |  |  |  |
| 10 | Oral herbal therapies for treating osteoarthritis | Reumalex vs placebo | AIMS2 arthritis pain score change from baseline | MD  -0.89 [-1.73, -0.05] | 7.84 | ***favours Reumalex*** |  |  |  |  |  |  |  |
| 11 | Oral herbal therapies for treating osteoarthritis | Boswellia serrata (enriched) 100 mg vs placebo | Pain (VAS) 0-100  90 days | MD  -16.57 [-24.67, -8.47] | 4.78 | ***favours Boswellia serrata*** |  |  |  |  |  |  |  |
| 12 | Exercise for osteoarthritis of the knee | Land-based exercise (Individual treatments) vs no exercise | Pain | SMD  -0.76 [-1.01, -0.52] | 3.97 | ***favours exercise*** |  |  |  |  |  |  |  |
| 13 | Oral herbal therapies for treating osteoarthritis | Salix purpurea x daphnoides vs diclofenac | Pain (WOMAC-VAS)  42 days | MD  15.0 [5.91, 24.09] | 3.50 | ***favours diclofenac*** |  |  |  |  |  |  |  |
| 13a | Oral herbal therapies for treating osteoarthritis | Salix purpurea x daphnoides vs diclofenac | Participants (n) reported adverse events | RR  0.63[0.43, 0.93] | 2.91 | ***Salix worse***  (CI : [1.20, 7.07]) |  |  |  |  |  |  |  |
| 14 | Oral herbal therapies for treating osteoarthritis | Persea gratissma + Glycine max (ASU 600 mg) vs placebo | Pain (VAS) 0-100 | MD  -14.2 [-20.82, -7.58] | 3.37 | ***favours ASU (600mg)*** |  |  |  |  |  |  |  |
| 15 | Acupuncture for hip osteoarthritis | Acupuncture vs NSAIDs | Pain | MD  -5.67 [ -10.11, -1.23] | 3.18 | ***favours acupuncture*** |  |  |  |  |  |  |  |
| 16 | Hyaluronic acid and other conservative treatment options for osteoarthritis of the ankle | Hyaluronic acid vs Placebo (progressive ankle exercise) | AOS total (combined pain & function score)  6 mo | MD  -12.53 [-23.84, -1.22] | 3.18 | ***favours hyaluronic acid*** |  |  |  |  |  |  |  |
| 19 | Acupuncture for hip osteoarthritis | Acupuncture vs sham acupuncture | Quality of Life | MD  -8.0 [-14.97, -1.03] | 2.78 | ***favours acupuncture*** |  |  |  |  |  |  |  |
| 21 | Oral or transdermal opioids for osteoarthritis of the knee or hip | Opioids versus placebo | Pain | MD  -0.28 [-0.35, -0.20] | 2.47 | ***favours opioids*** |  |  |  |  |  |  |  |
| 21a | Oral or transdermal opioids for osteoarthritis of the knee or hip | Opioids versus placebo | Withdrawal Symptoms | OR  2.76 [ 2.02, 3.77] | 2.76 | ***opioids worse***  (CI: [ 2.02, 3.77]) |  |  |  |  |  |  |  |
| 21b | Oral or transdermal opioids for osteoarthritis of the knee or hip | Opioids versus placebo | Participants (n) experiencing any adverse event | RR  1.49 [1.35, 1.63] | 3.00 | ***opioids worse***  (CI: [2.47 – 3.40]) |  |  |  |  |  |  |  |
| 22 | Oral herbal therapies for treating osteoarthritis | Zingiber officinale + Alpinia galanga (EV.EXT77) vs placebo | Pain (VAS) 0-100 after walking 50 ft | MD  -9.60 [-16.81, -2.39] | 2.43 | ***favours EV.EXT77*** |  |  |  |  |  |  |  |
| 23 | Exercise for osteoarthritis of the knee | Land-based exercise (Class-based programmes) vs no exercise | Pain | SMD  -0.42 [-0.51, -0.33] | 2.14 | ***favours exercise*** |  |  |  |  |  |  |  |
| 24 | Celecoxib for osteoarthritis | Celecoxib vs placebo | All pain  < 24 weeks | SMD  -0.41 [-0.49, -0.32] | 2.10 | ***favours celecoxib*** |  |  |  |  |  |  |  |
| 25 | Chondroitin for osteoarthritis | Chondroitin vs Placebo  ≥ 800 mg/d | Pain on a 0 to 100 scale ≥ 6mo | SMD  -0.39 [ -0.78, 0.00] | 2.03 | ***favours chondroitin*** |  |  |  |  |  |  |  |
| 26 | Exercise for osteoarthritis of the hip | Land-based exercise vs no exercise | Pain  3-6mo | SMD  -0.39 [-0.61, -0.17] | 2.03 | ***favours exercise*** |  |  |  |  |  |  |  |
| 27 | Exercise for osteoarthritis of the knee | Land-based exercise (Home programmes) vs no exercise | Pain | SMD  -0.38 [-0.55, -0.21] | 1.99 | ***favours exercise*** |  |  |  |  |  |  |  |
| 28 | High‐intensity versus low‐intensity physical activity or exercise in people with hip or knee osteoarthritis | High vs low intensity exercise | Pain (WOMAC) | MD  -1.33 [-2.56, -0.10] | 1.92 | ***favours high intensity*** |  |  |  |  |  |  |  |
| 29 | Chondroitin for osteoarthritis | Chondroitin sulfate or Chondroitin sulfate + Glucosamine vs Placebo or Control  ≥ 800 mg/d | Pain on a 0 to 100 scale | SMD  -0.65 [-0.95, -0.35] | 1.89 | ***favours chondroitin sulfate/chondroitin sulfate + glucosamine*** |  |  |  |  |  |  |  |
| 30 | Aquatic exercise for the treatment of knee and hip osteoarthritis | Aquatic exercise vs control (usual care, education, social attention, telephone call, waiting list for surgery) immediately after treatment: knee and hip OA | Pain  Immediately post treatment | SMD  -0.31 [-0.47, -0.15] | 1.75 | ***favours aquatic exercise*** |  |  |  |  |  |  |  |
| 31 | Exercise for osteoarthritis of the knee | Land-based exercise vs no exercise | Quality of life Immediately Post treatment | SMD  0.30 [0.04, 0.57] | 1.72 | ***favours exercise*** |  |  |  |  |  |  |  |
| 32 | Exercise for hand osteoarthritis | Exercise versus no exercise | Pain  (short term) | MD  -0.27 [-0.47, -0.07] | 1.63 | ***favours exercise*** |  |  |  |  |  |  |  |
| 33 | Aquatic exercise for the treatment of knee and hip osteoarthritis | Aquatic exercise vs control (usual care, education, social attention, telephone call, waiting list for surgery) immediately after treatment: **knee and hip OA** | Quality of life  (Immediately post) treatment | SMD  -0.25 [-0.49, -0.01] | 1.57 | ***favours aquatic exercise*** |  |  |  |  |  |  |  |
| 34 | Intra‐articular corticosteroid for knee osteoarthritis | Corticosteroid vs control (sham injection or no treatment) | Pain  3mo | MD  -0.22 [-0.44, 0.00] | 1.49 | ***favours corticosteroid*** |  |  |  |  |  |  |  |
| 35 | Self‐management education programmes for osteoarthritis | SMP versus usual care/no treatment/wait list | Pain  6wk-1yr | SMD  -0.17 [-0.26, -0.08] | 1.36 | ***favours SMP*** |  |  |  |  |  |  |  |
| Comments: | | | | | | | | | | | | | |

1. For Ease of Implementation: How easily can the intervention be added to usual care without requiring too much effort from health workers or disrupting practice. Consider how much effort is required, what training and resources are needed, what kind of scheduling and follow up, physical space, etc. Rankings are 0 to 4. 4= optimal (easier to implement), 0=more difficult [↑](#footnote-ref-1)
2. For Health System Effects: the impact the intervention will have on the health system. Certain interventions may be easy to implement but will still have large system effects – they may require incremental or major changes to the health system. 4= optimal (easier/fewer health system effects), 0=more difficult/greater health system effects, [↑](#footnote-ref-2)
3. For universality: Applicable and beneficial to large numbers including interventions targeted at one segment of the population (e.g. men) but the effects are wider than those targeted for the intervention (e.g. circumcision of men helps prevent HIV infection in women). An intervention targeted to all the PLHIV should be considered over for example MSM’s living with HIV who have TB and Hep C co-infection -  a very small specific group. Rankings are 0 to 4. 4= Optimal (more generalizable/population-based, 0= less generalizable/specific population

   ^4^ Does the distribution of the disease burden affect mainly the disadvantaged? Are the disadvantaged most likely to benefit from the intervention? Will the intervention improve equity in disease burden distribution long-term? Rank 0 for interventions that may increase inequities. Rank 4 for optimal interventions that would decrease inequities.

   ^5^ Are there any safety concerns related to the intervention that would impact your ranking? If so, please state them here. [↑](#footnote-ref-3)
