# Supplementary material for: Experts prioritize osteoarthritis non-surgical interventions from Cochrane systematic reviews for translation into “Evidence4Equity” summaries
Source: Int J Equity Health. 2021 Jun 10;20:136. doi: 10.1186/s12939-021-01477-4 (PMC8193871; doi:10.1186/s12939-021-01477-4)
Supplement: Supplementary file 2 — Additional file 2. [file 12939_2021_1477_MOESM2_ESM.docx]

Appendix B. Interventions grouped according to systematic review

| ID | Systematic Review | Intervention |
| --- | --- | --- |
| 1 | Acupuncture for hip osteoarthritis | Acupuncture + routine vs routine alone |
|  |  | Acupuncture vs sham acupuncture |
|  |  | Acupuncture vs NSAIDs |
| 2 | Aquatic exercise for the treatment of knee and hip osteoarthritis | Aquatic exercise vs control (usual care, education, social attention, telephone call, waiting list for surgery) for knee and hip OA |
| 3 | Braces and orthoses for treating osteoarthritis of the knee | Lateral wedge insole versus no insole |
|  |  | Brace vs no treatment |
| 4 | Celecoxib for osteoarthritis | Celecoxib vs placebo |
| 5 | Chondroitin for osteoarthritis | Chondroitin sulfate ( + glucosamine) vs placebo or control ≥ 800 mg/d |
|  |  | Chondroitin vs Placebo ≥ 800 mg/d |
| 6 | Exercise for hand osteoarthritis | Exercise vs no exercise for hand OA |
| 7 | Exercise for osteoarthritis of the hip | All land-based exercise vs no exercise for hip OA |
| 8 | Exercise for osteoarthritis of the knee | Home land-based exercise vs no exercise for knee OA |
|  |  | Individual Land-based exercise vs no exercise for knee OA |
|  |  | Class land-based exercise vs no exercise for knee OA |
| 9 | High‐intensity versus low‐intensity physical activity or exercise in people with hip or knee osteoarthritis | High vs low intensity exercise |
| 10 | Hyaluronic acid and other conservative treatment options for osteoarthritis of the ankle | Hyaluronic acid vs placebo + progressive ankle exercise |
| 11 | Intra‐articular corticosteroid for knee osteoarthritis | Corticosteroid vs sham injection/no treatment |
| 12 | Oral herbal therapies for treating osteoarthritis | Ayurvedic RA-II vs placebo |
|  |  | Harpagophytum procumbens vs diacerhein |
|  |  | Boswellia serrata, enriched (100 mg) + non-volatile oil vs placebo |
|  |  | Reumalex vs placebo |
|  |  | Persea gratissma + Glycine max (600 mg) vs placebo |
|  |  | Pinus pinaster (150 mL) vs placebo |
|  |  | SKI306X (1800mg) vs placebo |
|  |  | Boswellia serrata, enriched (100 mg) vs placebo |
|  |  | Salix purpurea x daphnoides vs diclofenac |
|  |  | Zingiber officinale + Alpinia galanga (EV.EXT77) vs placebo |
| 13 | Oral or transdermal opioids for osteoarthritis of the knee or hip | Opioids versus placebo |
| 14 | Self‐management education programmes for osteoarthritis | Self-Management Program vs usual care/no treatment/wait list |
